# Molecular Dynamics Simulation of Palmitate Ester Self-Assembly with Diclofenac

**DOI:** 10.3390/ijms13089572

**Published:** 2012-07-31

**Authors:** Roghayeh Abedi Karjiban, Mahiran Basri, Mohd Basyaruddin Abdul Rahman, Abu Bakar Salleh

**Affiliations:** 1 Department of Chemistry, Faculty of Science, UPM Serdang, Universiti Putra Malaysia, Selangor 43400, Malaysia; E-Mails: mahiran@science.upm.edu.my (M.B.); basya@science.upm.edu.my (M.B.A.R.); 2 Laboratory of Molecular Biomedicine, Institute of Bioscience, UPM Serdang, Universiti Putra Malaysia, Selangor 43400, Malaysia; E-Mail: abubakar@biotech.upm.edu.my; 3 Structural Biology Research Center, Malaysia Genome Institute, UKM Bangi, Selangor 43600, Malaysia

**Keywords:** palmitate ester, Tween80, diclofenac acid, self-assembly, molecular dynamics simulation

## Abstract

Palm oil-based esters (POEs) are unsaturated and non-ionic esters with a great potential to act as chemical penetration enhancers and drug carriers for transdermal drug nano-delivery. A ratio of palmitate ester and nonionic Tween80 with and without diclofenac acid was chosen from an experimentally determined phase diagram. Molecular dynamics simulations were performed for selected compositions over a period of 15 ns. Both micelles showed a prolate-like shape, while adding the drug produced a more compact micellar structure. Our results proposed that the drug could behave as a co-surfactant in our simulated model.

## 1. Introduction

Transdermal drug delivery offers many advantages, such as a continuous release into the blood over a longer time and the elimination of gastrointestinal difficulties [1]. The main problem is the skin barrier, which is a rigid lipid bilayer structure at the *stratum corneum*. Nanoemulsions, due to their very small droplet size and long-term physical stability, have been introduced as a potential solution to this problem. Nanoemulsions can increase the permeation of the drug in a number of diseases and disorders, such as Parkinson’s and Alzheimer’s diseases [2,3]. The nanoemulsion system consists of oil, surfactant, and water with a droplet size in the range of 20–200 nm [4]. The chemical constituents of the nanoemulsion system are considered to be chemical permeation enhancers (CPEs). Nanoemulsions of palm oil-based esters (POEs) have been suggested as potential CPEs due to their lipophilic properties [5].

POEs are unsaturated and nonionic esters that can be prepared by palm oil enzymatic synthesis. Several types of esters that can be found in POEs include oleyl laurate (C30:1) (0.4%), oleylmyristate (C32:1) (2.6%), oleyl palmitate (C34:1) (42.1%), oleyl stearate (C36.1) (5.3%), oleyl oleate (C36:2) (31.7%), and oleyl linoleate (C36:3) (10.2%) [6]. Each of these esters can contribute excellent physicochemical properties to the nanoemulsion system. The hydrophilic-lipophilic balance (HLB) value of POEs has not been reported, but the polar group at the head of POEs makes them hydrophilic. Palm oil-based nanoemulsions are described as dispersions of aggregated molecules of POEs and surfactant molecules in water [7]. In the formulation of nanoemulsions for transdermal drug nano-delivery, nonionic and nontoxic surfactants, such as Polysorbate 80 (or Tween80^®^), are usually used to reduce the interfacial tension between oil and water due to their amphiphilic properties [8]. Tween80 are odorless and tasteless with a hydrophilic-lipophilic balance (HLB) value of 15.0 ± 1.0 and are often used as an emulsifier, a solubilizer and a wetting agent [9]. By definition, the aggregation occurs above the critical micelle concentration (CMC) for a multicomponent system that follows Equation (1), where *C*_i_ is the CMC for each type of amphiphiles and *f*_i_ is the activity coefficient considering the nonideality of the solution [10].

(1) $$\frac{1}{CMC}=\sum_{i=1}^{n}\frac{x_{i}}{f_{i}C_{i}}$$

In recent years, molecular simulation techniques, such as molecular dynamics (MD), have shown very promising results for predicting micellar behavior in aqueous solution. Organizing virtual atoms using MD can effectively be used to simulate the molecular systems at the atomic level. MD calculations originate from selected force fields based on Newton’s equation of motion [11]. The all-atom level of MD, which is based on realistic potential energies, has been widely used to examine micelles with nonionic structures such as octyl glucoside [12,13], C_12_E_4_ in β-cyclodextrin [14], and dodecylphosphocholine (DPC) [15–17], in addition to ionic structures such as sodium dodecyl sulfate (SDS) [18] and sodium octanoate [19,20]. Abdul Rahman *et al.* (2009) reported that POE nanoemulsion could have a great potential to be used as a drug carrier for transdermal drug delivery [21]. A composition of water/POEs/Span20 (75:5:20) was chosen from the experimental phase diagram to perform MD simulation. From their results, a spherical micelle was formed where the POE molecules were surrounded by Span20. In another study by Abdul Rahman *et al.* (2010), a micellar system, consisting of oleyl oleate (OE) with nonionic Span20 (S20) and Tween80 (T80), was simulated in water to investigate the self-assembly profile of the micelles that formed [22]. The simulation results showed a cylindrical micelle for both models, while the OE/T80 system exhibited a lower eccentricity and a smaller size compared to the OE/S20 micelle. However, after 20 ns, the OE/T80 model also showed a spherical shape.

**Figure 1:**
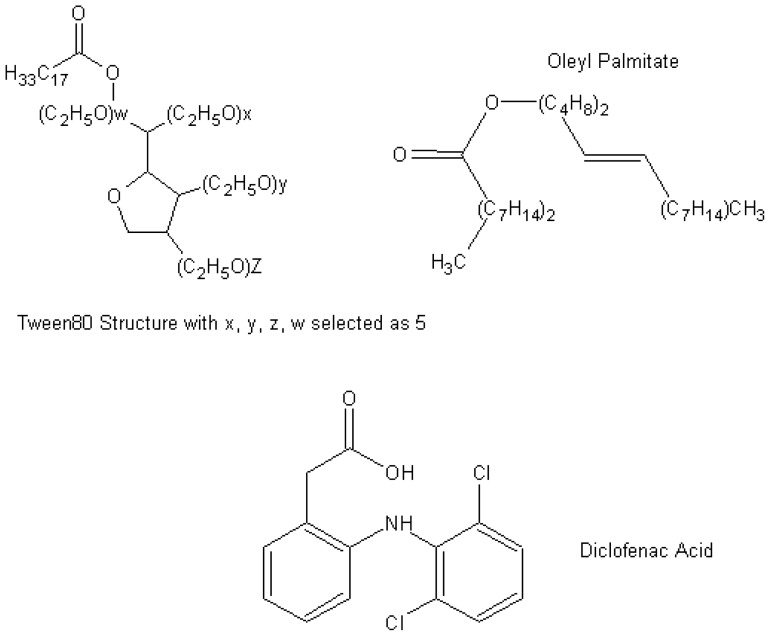
Chemical structure of oleyl palmitate, Tween80, and Diclofenac Acid.

Here, we aim to investigate the self-assembly of the micelles of oleyl palmitate (OP) and nonionic Tween80 (T80) with and without diclofenac acid (DIF) to understand the drug’s stability and its interaction with the nanoemulsion system (see Figure 1 for molecular structures). Two series of MD simulations were performed at different compositions of OP/T80/water and OP/T80/DIF/water according to the experimental formulation for 15 ns. Diclofenac acid or 2-[(2,6-dichlorophenyl)amino] benzene acetic acid (C_14_H_11_Cl_2_NO_2_) is a non-steroidal anti-inflammatory hydrophobic drug (NSAID) with the molecular mass of 296.148. It is commercially available in the form of anhydrous sodium salt. Diclofenac is used for the treatment of rheumatoid arthritis, osteoarthritis, ankylosing spondylitis, and a variety of non-rheumatic inflammatory problems. Gastrointestinal difficulties frequently occur after oral consumption for a long period of time, so delivery through the skin by using the nanoemulsion formulation will be a good alternative to decrease its adverse effects [23].

## 2. Results and Discussion

**Figure 2:**
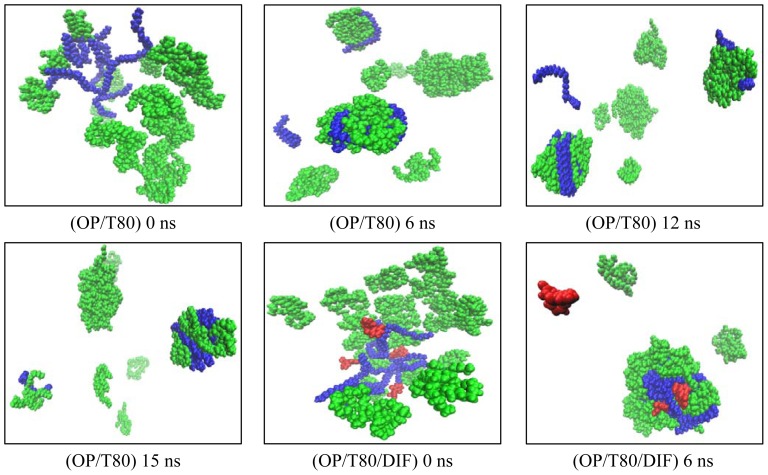

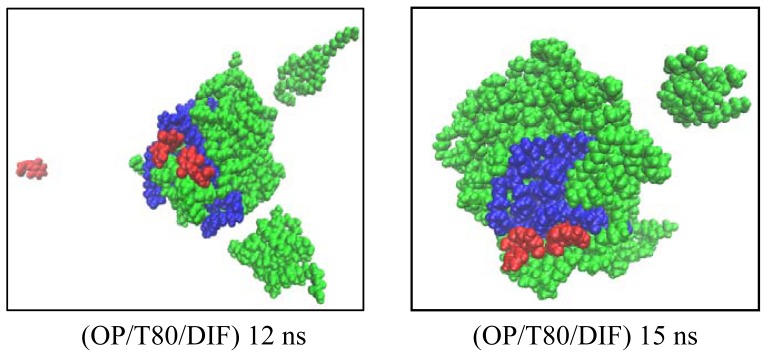
Snapshot pictures during molecular dynamics (MD) simulations of oleyl palmitate (OP)/Tween80 (T80) & OP/T80/diclofenac (DIF) systems at 300 K; OP (Blue); T80 (green); DIF (red).

The formation of micelles can be explained by the interaction of molecules/atoms with one another. We calculated the number of the clusters formed based on the distance between molecules by using the g_clustsize tool in GROMACS version 3.3.2 [24,25]. Molecules that were located over 0.5 nm away from one another were not considered to be part of the same cluster. Figure 2 shows snapshot pictures from our simulated models. As observed above, the OP molecules showed a great potential for self-assembly. This is because the hydrophobic tail of the ester can interact strongly with other ester molecules and with surfactant molecules [26]. Additionally, the molecules that were located at the surface of the smaller clusters tended to disengage themselves and to attach to each other to form the bigger clusters as a result of the Ostwald ripening [27]. This process was repeated many times until a stable aggregate formed. Although the molecules in OP/T80 model did not aggregate to form one large cluster, they moved aggressively throughout the simulation until several clusters were formed, and the number of clusters remained constant until the end of the simulation. The lower stability of the OP/T80 system in 15 ns may suggest that exchange with the bulk solution is a fast process for this model micelle [28].

From our results, adding DIF to the OP/T80 micellar system produced a stable micelle after 10 ns with few escaped OP and DIF molecules (see Figure 2). Our results for the OP/T80/DIF aggregated structure were in consistent with the study reported by the De Smet *et al*., (1999), where the rate of Ostwald ripening could be affected by the presence of oil-swollen surfactant micelles in the aqueous solution [29]. The aggregation of molecules to form a micelle can be influenced by both hydrophobic and hydrophilic effects. A system with amphiphilic behavior, such as POEs and nonionic Tween80 with a high HLB value, assists in the formation of a stable micellar system. At the same time, diclofenac may also behave as a co-surfactant supporting micelle formation.

It has already been shown that micellization is a dynamic process in which the micelles show a size distribution rather than a specific size [30]. The computation of the effective radius, *R*_s_, of a micelle is an excellent quantitative approach to compare the simulated values with the experimentally observed values [12]. If we assume that the micelles are an approximately spherical shape, the effective radius of the micelle depends on the radius of a solid sphere with uniform density. The effective radius can be calculated from the average radius of gyration according to the following equation:

(2) $$R_{s}=\sqrt{5/3}≺R_{g}≻$$

**Figure 3:**
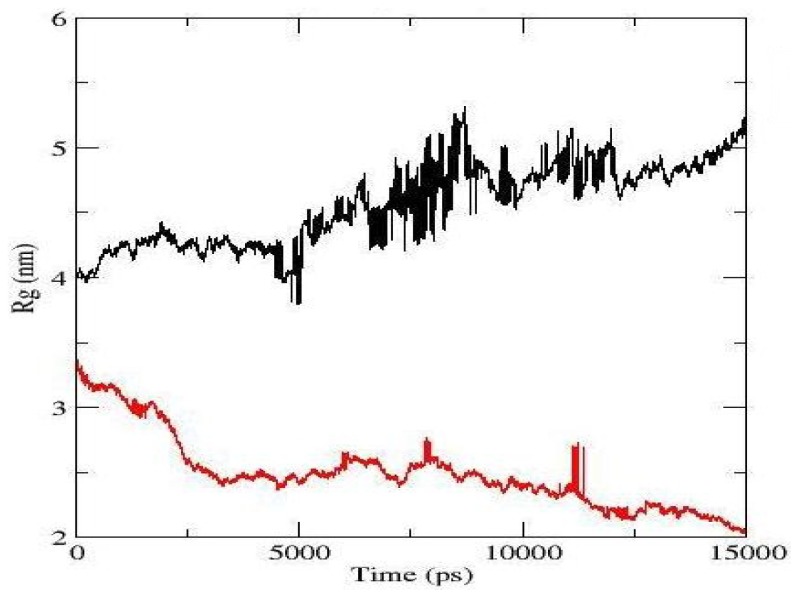
The radius of gyration, with fluctuation as a function of time for both simulations; black (OP/T80) and red (OP/T80/DIF).

Figure 3 shows the fluctuation in the radius of gyration, *R*_g_, as a function of time for both simulated models [black for the OP/T80 system and red for the OP/T80/DIF model]. The compactness of the micelles can be estimated from the *R*_g_ values. From Figure 3, a sharp decrease in *R*_g_ was observed for the OP/T80/DIF model until 2.5 ns, and then it smoothly decreased until the end of the simulation. However, some sudden spikes were observed approximately 8 ns and 11.5 ns that were most likely a result of periodic boundary conditions. In the OP/T80 model, the *R*_g_ increased for the first few picoseconds of the simulation, and then it remained constant until 5 ns. After 5 ns, it constantly increased until the end of the simulation. However, several spikes were observed between 5 ns and 12 ns. After 12 ns, both models showed a constant fluctuation in the radius of gyration until the end of the simulations with a tendency to increase for OP/T80 and decrease for OP/T80/DIF. As observed below, our *R*_g_ results showed the formation of one stable aggregate in the OP/T80/DIF model, which can also be observed in Figure 2.

Table 1 reports a summary of the effective radius of the micelles formed and the average radius of gyration in addition to the eccentricity value and the moment of inertia along either the *x*, *y*, or *z* axes. All calculations were averaged over the last 500 ps of the production simulations. The escaped molecules were not included in the subsequent calculations. Our results showed that the OP/T80/DIF micelle was more compact compared to the OP/T80 micelle. The shape of the micelles formed can be quantified by monitoring the three time-averaged principle moments of inertia and evaluating the eccentricity value, *e*, from Equation (3) [31],

(3) $$e=1-I_{\text{min}}/I_{avg}$$

where *I_min_* is the moment of inertia along either the *x*, *y*, or *z* axis with the smallest value, and *I_avg_* is the average value over all three axes. The moment of inertia was calculated from the gyration results according Equation (4), where *m_i_* is the mass of the atom *i*, and *r_i_* is the distance between the atom and the center of mass of each molecule involved.

(4) $$R_{g}={\left(\frac{\sum_{i}r_{i}^{2}m_{i}}{\sum_{i}m_{i}}\right)}^{1/2}$$

The micelle with an eccentricity value of zero showed a perfect spherical shape; however, our eccentricity results did not suggest the formation of any spherical micelles (see Table 1). We estimated the shapes of the micelles formed by calculating the ratio of the average principal moments of inertia tensors. This analysis is particularly important when the micelle shows significant deviation from the spherical/symmetrical shape [32]. The estimated ratios of the principal moments of inertia were 1.5:1.4:1 for the OP/T80 system and 1.3:1.1:1 for the OP/T80/DIF model; these ratios mimic a prolate-like shape. The values for the OP/T80 model system, however, were much further apart.

The solvent accessible surface area (SASA) can also be used to quantitatively determine the molecular packing in the micellar system. The water accessibility of the surfactant can be evaluated via estimation of the SASA value per molecule of surfactant depending on the micelle size. Table 2 reports the average hydrophobic and hydrophilic values and the total SASA value for our simulated models. A higher total SASA value of 221.52 (±5.33) nm^2^ per surfactant was found for the OP/T80 model compared to 153.65 (±2.57) for the OP/T80/DIF system; these results indicate a slightly more diffuse and irregular arrangement of molecules relative to each other. The SASA value of the hydrophobic moieties showed the same trend for both models; this result implies that the surfactant molecules packed rather tightly against the surface at the core. This observation might be related to the fact that the micelle geometry re-arranged upon the addition of the DIF molecules. As observed from the results, the area of the micelle surface covered by the hydrophilic SASA decreased only slightly for the two simulated systems, while the area of the hydrophobic parts decreased significantly. As such, the surfaces of the OP/T80 micelle were more hydrophilic, as expected, due to the strong interactions between the surfactant and the water molecules.

Diclofenac is topically administered in the form of gel as 1% sodium salt because it has a high partition coefficient (log P = 4.0) and very low water solubility (17.8 mg/L) in the unionized form [23]. Furthermore, due to several adverse effects, such as a high portion of hepatic first-pass metabolism (~50%) as well as a short biological half-life (1.2–2 h), diclofenac is administered frequently. Therefore, the delivery of diclofenac through the skin may provide better patient compliance over oral consumption [33]. The high hydrophobicity of the molecule is partly maintained even when the drug is in the form of salt. The Diclofenac anion has a positive log P of 0.69, as calculated by the fragment constant method [34]. Diclofenac salt is soluble in aqueous solution, and its penetration into the skin depends on partitioning of the unionized form into the lipophilic phase of the topical emulsion [35]. Although diclofenac acid is a hydrophobic molecule with low solubility in water due to its structure, the hydrophilic carboxylic acid plays an important role in assisting the aggregation process.

**Figure 4:**
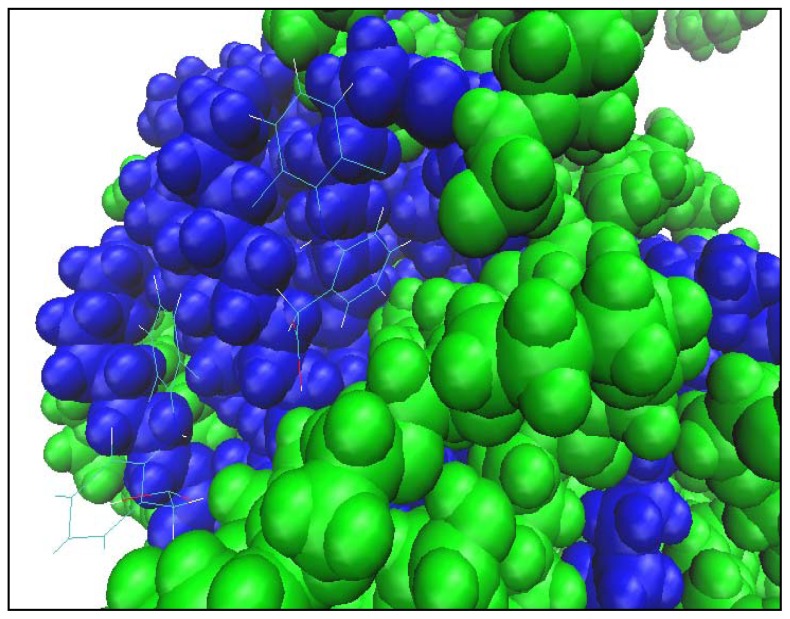
Close-up of a typical conformation of diclofenac molecules (upper) and of oleyl palmitate and Tween80. For the basic color scheme, see caption of Figure 2. Additionally, all oxygens were colored red and hydrogens of the hydroxyl groups white to illustrate the hydrogen bonds.

The hydrophobic properties of diclofenac will lead the drug molecules to the micelle surface; this effect is the same as what occurs with the hydrophobic Ibuprofen molecule. Long *et al.*, (2006) simulated the solid lipid microparticles (SLM) loaded with Ibuprofen molecules and examined the drug release for the SLM carrier [36]. They found that the Ibuprofen molecules were mainly distributed on the surface of the SLM micelle until the end of the simulation, which was a period of 10 ns. From our results, diclofenac molecules were similarly placed at the surface of the micelle (see Figure 4).

Tween80 and palmitate ester possess the hydrophilic hydroxyls that can easily form hydrogen bonds between these molecules and the carboxyl group of diclofenac. Thus, the hydrophobic tail of the diclofenac remains in the body of the carrier with the hydrophilic part at the oil/water interface along with the Tween80 and palmitate ester molecules. However, Tween80 was used as a surfactant in our study, but due to the characteristic properties of diclofenac molecule, we suggest that diclofenac may have behaved as a co-surfactant in the self-assembly process.

## 3. Experimental Section

MD simulations were performed based on the OPLS-AA force field [37], and the oleyl palmitate (OP), Tween80 (T80), and diclofenac (DIF) atom types were assigned accordingly. All structures were geometry-optimized by using WinGAMESS [38]. Due to the large size of the T80 and OP molecular structures, optimization was first performed at a lower basis set of STO-3G, and then the equilibrated geometries were re-optimized at the 6-31G level, while the DIF optimization was directly performed at the 6-31G level. The atomic charges were then computed using the Restrained Electrostatics Potential (RESP) method based on electrostatic potentials (ESP) calculated at the 6-31G level in all structures [39].

MD simulations and energy minimization were performed using GROMACS version 3.3.2 [24,25]. All starting structures were placed in a cubic simulation box with pre-equilibrated SPC water [40]. The prepared systems were then energetically minimized using the steepest descent and conjugate gradient techniques until the minimization converged with the maximum force (less than 5 kJ mol^−1^ nm^−1^). The systems were heated for 20 ps at 300 K in an NVT ensemble using periodic boundary conditions. A 2 fs stepsize was used with all covalent bonds to hydrogen atoms held rigid using the LINCS approach [41], while the water geometry was constrained using the SETTLE algorithm [42]. The temperature was kept constant at 300 K with τ_T_ = 0.5 using the Berendsen thermostat [43]. The electrostatic interactions were cut-off at 1.0 nm using the Particle-Mesh-Ewald (PME) method [44]. Fourth-order interpolation was applied by setting PME_order = 4. This spacing gave electrostatic energies accurately to approximately 5 × 10^−3^. The van der Waals interactions were cut-off at 1.4 nm for the OP/T80 system, while the cut-off distance for the system involving DIF (OP/T80/DIF) was set at 1.0 nm. The neighbor-searching was cut off at 1.0 nm. To obtain an equilibrated structure, a short equilibration of 300 ps followed by a production of 1 ns with a step size of 2 fs in an NPT ensemble was carried out with the same protocols, except the van der Waals interactions were cut-off between 1 and 1.2 nm for all molecules. The pressure was coupled with the Parrinello-Rahman thermostat at 1.0 bar with a τ*_P_* of 1.0 [45].

The ternary phase diagram of OP/T80/water was experimentally determined by our research group [46]. We chose one spot from the isotropic region (20:50:30; OP:T80:W) for our simulations. The weight-to-weight ratio of OP/T80 was divided by the molecular weight of each component and was then multiplied by Avogrado’s number to obtain the total number of molecules for each simulated model. Two model systems were prepared. The composition of molecules in our first model without inserting DIF (OP/T80) was chosen as OP (8) and T80 (20). For another model with the drug (OP/T80/DIF), 4 molecules of DIF were added to the previous system. To form the initial mixtures, the equilibrated T80, OP, and DIF molecules were chosen. Twenty molecules of T80 and 8 molecules of OP were placed randomly in a cube using Packmol [47] for OP/T80 model, and then 4 molecules of DIF were added for the OP/T80/DIF model system, followed by salvation in a pre-equilibrated system of SPC water. Two series of MD simulations were performed (see Table 3). Both systems were first energetically minimized using both the steepest-descent and conjugated-gradient techniques and then equilibrated for 300 ps. MD production simulations were performed in an NPT ensemble with a stepsize of 2 fs for 15 ns. The MD protocols were the same as above except that the temperature was coupled to a 300 K bath with the τ_T_ changing between 0.2 and 0.5, and the van der Waals forces were cut-off at 1.0 nm.

## 4. Conclusions

We carried out all-atom MD simulations of oleyl palmitate ester and Tween80 micelle formation with and without diclofenac in aqueous solution for 15 ns. Our results revealed that the self-assembly of these model systems from randomly positioned molecules into micelles is fast and can occur within a nanosecond time scale. The structural properties that we estimated revealed a prolate-like shape for both model systems, and adding the drug produced a more compact micellar structure. Additionally, the drug behaved as a co-surfactant in our simulated models. The presented theoretical study gave a more detailed insight into understanding the complexity of the aggregation process in palm oil-based nanoemulsions and their applications as chemical penetration enhancers and drug carriers.

## Figures and Tables

**Table 1:** A Summary of the averaged *R*_g_, *R*_s_, moment of inertia, and eccentricity values of the OP/T80 and OP/T80/DIF models for the last 500 ps of both simulations.

Model system	<*R*_g_> (nm)	*R*_s_ (nm)	<I_1_> (10^4^ amu nm^2^)	<I_2_> (10^4^ amu nm^2^)	<I_3_> (10^4^ amu nm^2^)	I_1_:I_2_:I_3_	*e*	Droplet size (Exp.) (nm)
OP/T80	5.10 (±0.006)	6.58	60.22 (±1.24)	55.80 (±1.10)	39.94 (±1.96)	1.5:1.4:1	0.6	5.87
OP/T80/DIF	2.07 (±0.002)	2.67	10.43 (±0.31)	8.75 (±0.15)	7.86 (±0.25)	1.3:1.1:1	0.5	-

**Table 2:** A summary of the hydrophobic, hydrophilic, and total solvent accessible surface area (SASA) values of three model systems, averaged per molecule of Tween80 for the last 500 ps of both simulations.

Model system	Hydrophobic (nm^2^)	Hydrophilic (nm^2^)	Total (nm^2^)
OP/T80	192.58 (±4.65)	28.94 (±0.93)	221.52 (±5.33)
OP/T80/DIF	132.41 (±2.23)	21.24 (±0.91)	153.65 (±2.57)

**Table 3:** An overview of the MD simulations.

Model	Number of molecules	# SPC	Box edge (nm)	Average density (g/cm^3^)	Concentration (w%)	Average total energy (kJ/mol)
T80	1	5368	5	1.01 ± 0.00	1.4	−1.69 × 10^5^ ± 305
OP	1	2145	4	1.01 ± 0.00	1.3	−6.76 × 10^4^ ± 191
DIF	1	1391	3.5	1.01 ± 0.00	1.2	−4.41 × 10^4^ ± 142
OP/T80	20;8	49133	11.5	-	3.0;0.5	−1.52 × 10^6^ ± 1140
OP/T80/DIF	20;8;4	55837	12	-	2.6;0.4;0.1	−1.74 × 10^6^ ± 1038
